# Identification of binding pockets in protein structures using a knowledge-based potential derived from local structural similarities

**DOI:** 10.1186/1471-2105-13-S4-S17

**Published:** 2012-03-28

**Authors:** Valerio Bianchi, Pier Federico Gherardini, Manuela Helmer-Citterich, Gabriele Ausiello

**Affiliations:** 1 Centre for Molecular Bioinformatics, Department of Biology, University of Rome "Tor Vergata", Via della Ricerca Scientifica snc, Rome 00133, Italy

## Abstract

**Background:**

The identification of ligand binding sites is a key task in the annotation of proteins with known structure but uncharacterized function. Here we describe a knowledge-based method exploiting the observation that unrelated binding sites share small structural motifs that bind the same chemical fragments irrespective of the nature of the ligand as a whole.

**Results:**

PDBinder compares a query protein against a library of binding and non-binding protein surface regions derived from the PDB. The results of the comparison are used to derive a propensity value for each residue which is correlated with the likelihood that the residue is part of a ligand binding site. The method was applied to two different problems: i) the prediction of ligand binding residues and ii) the identification of which surface cleft harbours the binding site. In both cases PDBinder performed consistently better than existing methods.

PDBinder has been trained on a non-redundant set of 1356 high-quality protein-ligand complexes and tested on a set of 239 holo and apo complex pairs. We obtained an MCC of 0.313 on the holo set with a PPV of 0.413 while on the apo set we achieved an MCC of 0.271 and a PPV of 0.372.

**Conclusions:**

We show that PDBinder performs better than existing methods. The good performance on the unbound proteins is extremely important for real-world applications where the location of the binding site is unknown. Moreover, since our approach is orthogonal to those used in other programs, the PDBinder propensity value can be integrated in other algorithms further increasing the final performance.

## Background

Many proteins carry over their function by interacting with small molecule ligands. These include enzyme cofactors, metabolites and chemical messengers such as hormones. Moreover the vast majority of drugs are in fact small molecules that bind to a target protein thus modulating its function. Therefore the identification of a ligand binding site can give indication about the molecular function of a protein, even though the relationship between the molecular and biological function, i.e. the role of a protein in the context of a cellular process, is not straightforward.

The task of predicting a binding site for a specific ligand can be divided in two steps: i) the identification of an appropriate cavity in the structure, ii) the prediction of which molecule can fit in said cavity. The latter task, which can be broadly identified with molecular docking, is extremely demanding from a computational point of view. Therefore the first step is almost always necessary in order to limit the search space of a molecular docking experiment.

Accordingly a variety of algorithms have been developed to identify ligand binding pockets in protein structures [1]. The available methods use geometric criteria, energy functions or a combination of both.

Geometric approaches, such as SURFNET [2], LIGSITEcsc [3], ConCavity [4], APROPOS [5] and DEPTH [6] mainly work by identifying cavities on the structure and then predicting one of the largest clefts as the ligand binding site. Indeed it has been shown with SURFNET that considering the two largest cavities calculated by this method identifies the correct location of the ligand in single-chain enzymes with a success rate of about 90% [7].

LIGSITEcsc combines geometric analysis with evolutionary conservation by ranking clefts according to the conservation of their constituent residues. The method was tested on 48 apo/holo protein pairs and on a second test set composed of 210 holo protein structures and correctly predicts the ligand binding site in 71% and 75% cases, respectively.

ConCavity also integrates sequence conservation with pocket detection and makes specific predictions of positions in space that overlap ligand atoms. Moreover, instead of identifying entire clefts, the method specifically predicts which residues are likely to be in contact with a ligand. ConCavity was tested on 332 apo/holo protein pairs from the non redundant LigASite 7.0 dataset [8] and achieved an area under the precision-recall curve of 0.61 and 0.66 on apo and holo structure respectively.

APROPOS uses an alpha-shape algorithm to identify pockets by comparing surfaces of the protein generated with different levels of detail. This algorithm correctly locates more than 95% of the ligand binding sites in a set of about 300 protein structures. DEPTH is based on the observation that some of the residues in a binding site could be deep but at the same time exposed to the solvent. DEPTH has been compared with LIGSITE, Pocket-Finder, SURFNET and Concavity on a set of 225 protein structures binding small ligands, achieving results comparable with all these methods.

Methods based on energetic criteria, such as the one developed by Morita *et al*. [9], Q-SiteFinder [10] and SITEHOUND [11], usually calculate the energy of interaction between a probe and the surface of the protein. Clusters of points with favorable interaction energies define the predicted ligand binding sites.

SITEHOUND uses an energy-based approach to identify possible regions of binding, implementing different types of probes (a carbon probe and a phosphate probe) to characterize a protein structure. It has been tested on 77 protein structures and it correctly ranks the ligand binding site in one of the top three clusters in 95% of the cases.

Both Q-SiteFinder and the method by Morita *et al*. use methane probes and have been tested on the same dataset of 35 apo/holo protein pairs. Q-SiteFinder achieves a success rate of 51% for apo and 80% for holo structures, when considering the top scoring prediction as the correct one. The method by Morita *et al*. has a similar performance on holo structures, but performs better on apo proteins achieving a success rate of 77%.

In addition to these approaches, a variety of methods exist that predict the location of binding sites based on several characteristics that distinguish them from other regions of the protein surface. For instance residues in binding pockets and enzyme active sites have been observed to interact directly, or via few intermediates, with a great number of residues in the structure [12]. Moreover enzyme active sites have been reported to have perturbed pKa values [13] and, when mutated, lead to a decrease in activity accompanied by an increase in the stability of the protein [14]. The preference of certain residues to be located in binding sites has also been exploited for prediction. Mehio *et al*. [15] recently developed a binding site prediction method based on the over-representation of specific atom triplets in binding sites with respect to the rest of the structure.

A separate discussion is necessary when the structure of a homologue of the protein of interest is available. For these cases different approaches have been proposed in the last few years, such as 3DLigandSite [16], FINDSITE [17] and firestar [18].

3DLigandSite searches a structural library for proteins homologous to the query. These proteins are superimposed with the structure of interest and the position of their ligands is used to predict the location of the binding site. Similarly FINDSITE superimposes and clusters ligands from homologous structures onto a query protein and uses the center of mass of the ligands to identify putative binding sites. The predicted sites are then ranked according to the number of templates sharing the binding pocket. Firestar integrates FireDB, a database of annotated functional residues, with a sequence alignment tool in order to enable the comparison of binding residues across homologous proteins. When possible the information of which residues are functionally important is transferred to the protein of interest.

As previously stated the performance of binding site prediction methods differs according to whether the analysis is performed on apo or holo structures because proteins undergo conformational changes when binding their cognate ligands. In general most of these methods correctly identify the location of the binding site in 70-90% of the cases if the protein analyzed is in the bound conformation (holo). In contrast, the same analysis performed on the apo structures achieves a success rate ranging from 50% to 70%.

A wealth of data concerning the mechanisms underlying the interaction of proteins with small molecules can be extracted from complexes of known structure deposited in the PDB. While knowledge-based approaches have been developed that use these data to dock ligands onto proteins [19-21], no method exists that uses available protein-ligand complexes to predict binding sites on a query structure, irrespective of the ligand.

In this work we detail the development of PDBinder, a novel method for the prediction of ligand binding sites in protein structures. PDBinder is a knowledge-based method based on the observation that unrelated binding sites share small structural motifs that bind the same chemical fragments irrespective of the nature of the ligand molecule as a whole [22]. Moreover these motifs behave as small building blocks (modules) that can be assembled to create different binding pockets [23]. This modularity parallels the modularity of the ligand molecules, which are themselves composed of recurring chemical fragments.

We therefore reasoned that, because of the presence of these motifs and irrespective of the identity of the bound ligand, a binding site should resemble other known binding sites, more than other parts of the structure. Thus the number of similarities that are identified between a protein patch and the set of all known binding sites can be used to assess the likelihood of that surface area being able to bind a ligand.

PDBinder uses the Superpose3D [24,25] local structural comparison algorithm to scan a query protein against a library of binding and non-binding protein surface regions derived from the PDB. The number of similarities identified in the two sets is then used to derive a propensity value for each residue in the protein of interest. Surface areas with high propensity values denote the position of the predicted binding site. The method was trained on a non-redundant set of 1356 high-quality structures of protein-ligand complexes and tested on two different datasets of 239 and 35 apo/holo structure pairs. We also report a comparison between the performance of PDBinder and other methods.

### Availability

A downloadable version of the program is available at http://cbm.bio.uniroma2.it/pdbinder/.

## Results and discussion

### Training of the method

**Figure 1:**
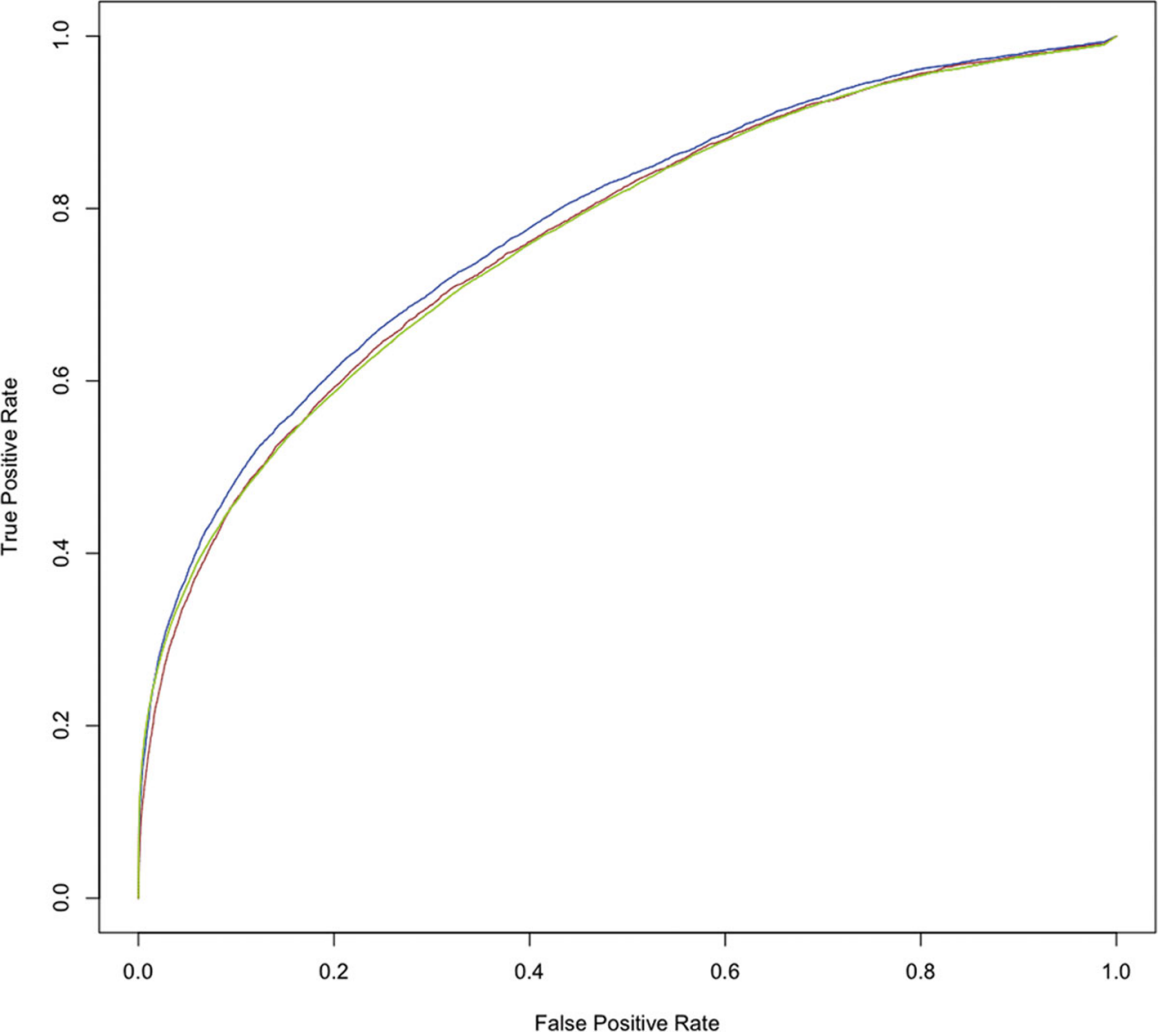
**ROC curves for the analysis on the Training Set and Apo/Holo Test Set**. Green: Training Set (AUC 0.766); blue: Holo Test Set (AUC 0.780); brown: Apo Test (AUC 0.767).

In order to train PDBinder and determine the optimal threshold of the propensity value we used the non-redundant dataset of 1356 high-quality ligand binding structures from which we derived the libraries of binding and non-binding residues. We used a leave-one-out procedure as described in the methods. As a first test we pooled the residues from all the structures together, each one associated with the PDBinder propensity value and a binary flag indicating whether the residue is part of a binding pocket or not. To assess the predictive power of the propensity value we drew a ROC curve and obtained an Area Under the Curve (AUC) of 0.765 (see Figure 1). This result shows that the propensity value assigned by PDBinder is indeed correlated with the likelihood that a residue is part of a binding pocket.

**Figure 2:**
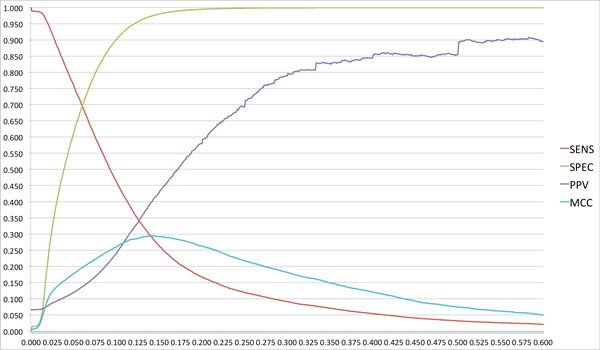
**Performance of PDBinder**. Sensitivity, Specificity, Positive Predictive Value and Matthew's Correlation Coefficient calculated as a function of the Propensity Value threshold.

In order to calculate the optimal propensity threshold we choose the value that maximizes the average of the prediction performance on all the proteins, i.e. instead of pooling all the residues together we calculate the performance for each chain and seek to maximize the average. This situation is clearly more indicative of the typical usage of the method. Figure 2 shows how the Sensitivity, Specificity, Positive Predictive Value (PPV) and Matthew's Correlation Coefficient (MCC) vary as a function of the threshold of propensity value. This figure also highlights the effects of using a threshold that maximizes the Positive Predictive Value, i.e. a threshold value of 0.584. The PPV rises to 0.907 but the Sensitivity decreases to 0.023 which means that only 2% of the binding pockets residues are correctly recognized.

We choose the threshold value that maximizes the average of the Matthew's Correlation Coefficient (MCC).

The optimal threshold was thus determined to be 0.143. Because of the way the PDBinder propensity is defined this means that at least ~15% of the matches of a residue must be with residues in binding pockets for the amino acid to be predicted as positive. Using this threshold resulted in an average MCC on the training set of 0.294, with an average sensitivity of 0.283, a specificity of 0.976 and a positive predictive value of 0.407.

### Clustering of the predictions

The results on the training set show that using the propensity value alone we obtain ~40% of correct predictions. Since binding sites are contiguous regions of the surface, we investigated whether discarding spatially isolated predictions yielded an improvement in the performance of PDBinder. We therefore decided to discard all the predictions that do not have at least another prediction in a defined radius. We tested different thresholds from 1.0 Å to 20.0 Å in increments of 1 Å. The results of this spatial filtering show that the best clustering radius is 10.0 Å. This value is close to the mean radius of the binding sites in the training set (8.97 ± 0.20 Å, 99% confidence interval). The use of this threshold raises the positive predictive value by 5.4% and the MCC by 0.018 with a loss of 1.4% in sensitivity (see Table 1).

**Table 1:** Benchmark results

	SENSITIVITY	SPECIFICITY	PPV	MCC
**TRAINING SET**	0.269	0.984	0.461	0.312
**HOLO TEST SET**	0.295	0.983	0.413	0.313
**APO TEST SET**	0.251	0.984	0.372	0.271

Results obtained by PDBinder on the Training Set and Apo/Holo Test Sets. These results were derived by using a single propensity threshold for all the amino acids and applying the spatial clustering with a 10 Angstrom threshold.

### Amino acid-specific analysis

The amino acid composition of binding pockets is known to be different from that of the remainder of the structure. Indeed some amino acids such as arginine and histidine are commonly found in ligand binding sites [26]. Therefore structural motifs including these residues will tend to match more frequently with binding sites by virtue of their composition alone. We tried to separate this effect from the contribution given by geometry by calculating a different propensity value threshold for each amino acid.

This method implicitly normalizes for the preference of certain amino acids to appear in binding sites. For instance, in order for the propensity of a histidine to rise above the threshold, the structural motifs of which it is part have to appear in known binding sites more frequently than other motifs involving histidine. If we use the same threshold for all the amino acids, the same histidine has to "compete" with all the motifs, which is easier because histidine is itself preferred in binding sites while the other motifs include residues which are strongly disfavored.

**Figure 3:**
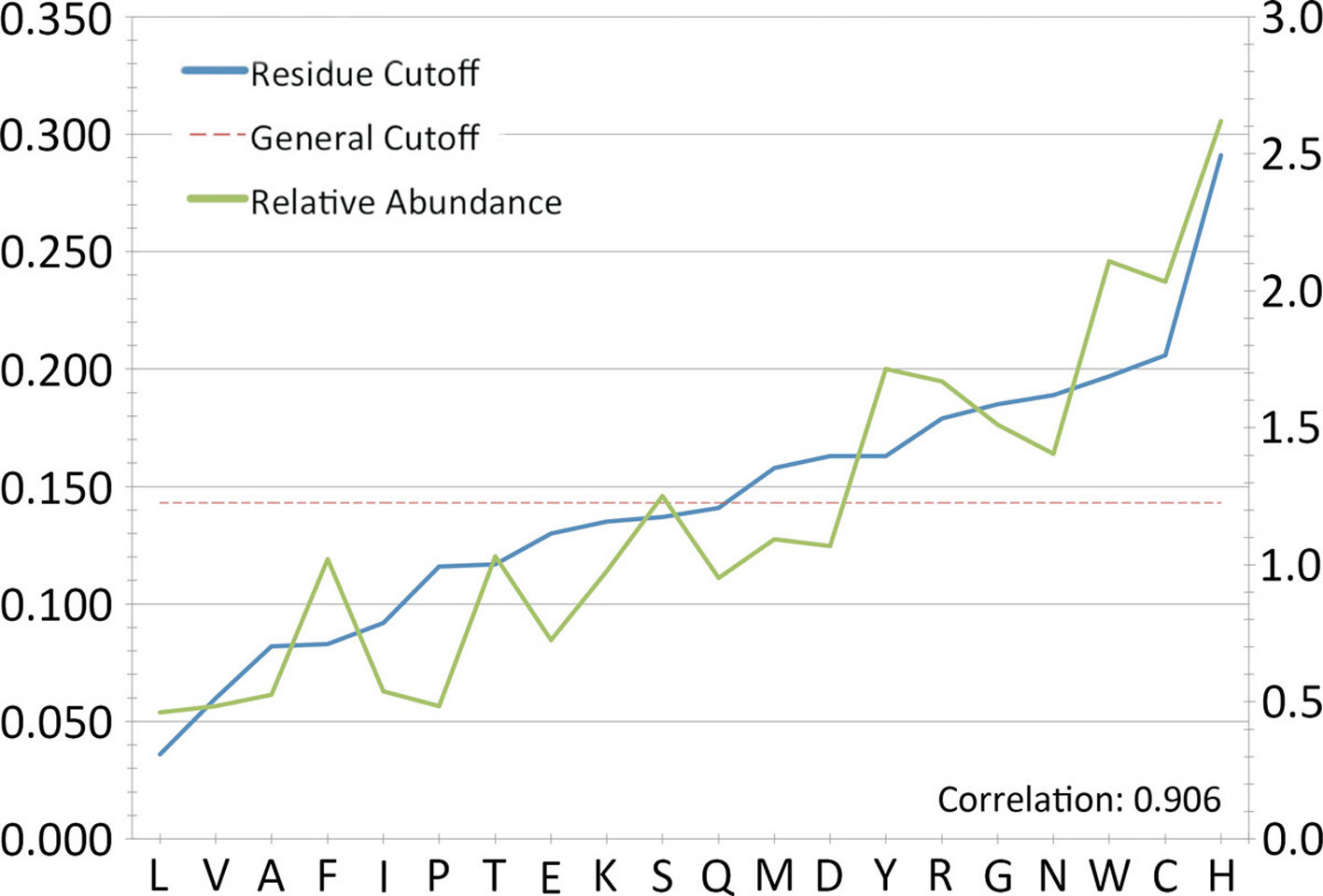
**Residue-specific cutoffs vs residues abundance**. The residue-specific cutoffs of propensity values are shown together with the abundance of each residue in binding sites. The abundance is calculated as the ratio between the relative abundance of each residue in binding and non-binding regions. The horizontal red line marks the general cutoff value (0.143) derived from the whole analysis. The Pearson's correlation coefficient between the two series is shown.

We therefore calculated 20 different thresholds using all the residues in the training set, with the same method that was used to derive the general cutoff (see above). Figure 3 displays the residue-specific cutoffs along with the general cutoff of 0.143 used in the previous analysis (red line). The optimal cutoffs are specific for each aminoacid type and range from the 0.036 of leucine to the 0.291 of histidine. Interestingly these cutoffs correlate well (Pearson's correlation coefficient 0.9) with the residue preferences calculated from the composition of the binding sites alone, with residues having a threshold higher than the general cutoff being favored.

We again calculated the average MCC for all the proteins in the training set, this time using the amino acid-specific cutoffs and obtained an MCC of 0.291 with an average sensitivity of 0.244 a specificity of 0.985 and a positive predictive value of 0.447. Therefore the use of residue-specific thresholds yielded fewer, more accurate predictions but the average performance was slightly worst and was not recovered by introducing the spatial clustering (data not shown).

We decided to determine how much the geometry of the binding residues is important for our knowledge-based potential with respect to the simpler aminoacid composition of the binding triplets. To this end we calculated an average propensity value for all the binding triplets of residues having the same aminoacid composition, i.e. AVV, VAV and VVA, regardless of their geometric configuration. To derive the average propensity value, we calculated the ratio between the number of times each triplet with the same aminoacid composition appears in a binding pocket and the total number of occurrences of the triplets having the same aminoacid composition.

The complete list of triplets along with their potentials is reported in the Additional file 1: Table 1.

We then applied these propensity values in a leave-one-out experiment on the training set. For each protein structure, the propensity value associated to a residue is calculated as the average propensity value of the triplets in which each residue is found using Superpose3D.

When using this simpler aminoacid composition potential, the best MCC obtained on our training set drops to 0.099 (corresponding to a propensity threshold of 0.254), thus showing the importance of the geometry of a triplet in determining whether a residue is binding or not.

These results show that geometry alone gives a strong contribution to the predictive power of PDBinder. However the signal from the binding site composition, i.e. the differential occurrence of specific residues, is too strong to be ignored without some loss of performance. The single cutoff method was therefore used for all the remaining analyses.

### Evaluation of the results with the test set

A benchmark dataset for binding site prediction methods should ideally consist of proteins with one unbound structure to apply the method, and at least one bound structure to assess the correctness of the predictions. This is necessary to account for the fact that proteins can undergo conformational changes upon binding. Consequently applying a binding site prediction method to a bound structure from which the ligand has been deleted does not appropriately reproduce situations where the binding site location is truly unknown.

For these reasons we tested PDBinder on the LigASite dataset, which includes, for each protein-ligand complex (holo structure), a structure determined in absence of the ligand (apo). The training and test sets are completely independent. Moreover, in order to avoid any unfair advantage deriving from the possible homology of a structure in the test set with the chains used to build the libraries of binding and non binding residues, we did not consider structural matches with residues coming from proteins having a sequence identity with the query structure higher than 30%.

We used the optimal propensity threshold of 0.143 which was derived from the analysis of the training set as described above. The average MCC value on the holo test set was 0.313 with an average sensitivity of 0.295, an average specificity of 0.983 and a PPV of 0.413 (see Table 1). The small decrease in performance shows that the optimal threshold derived from the training set is general enough to be applied to an independent dataset.

**Figure 4:**
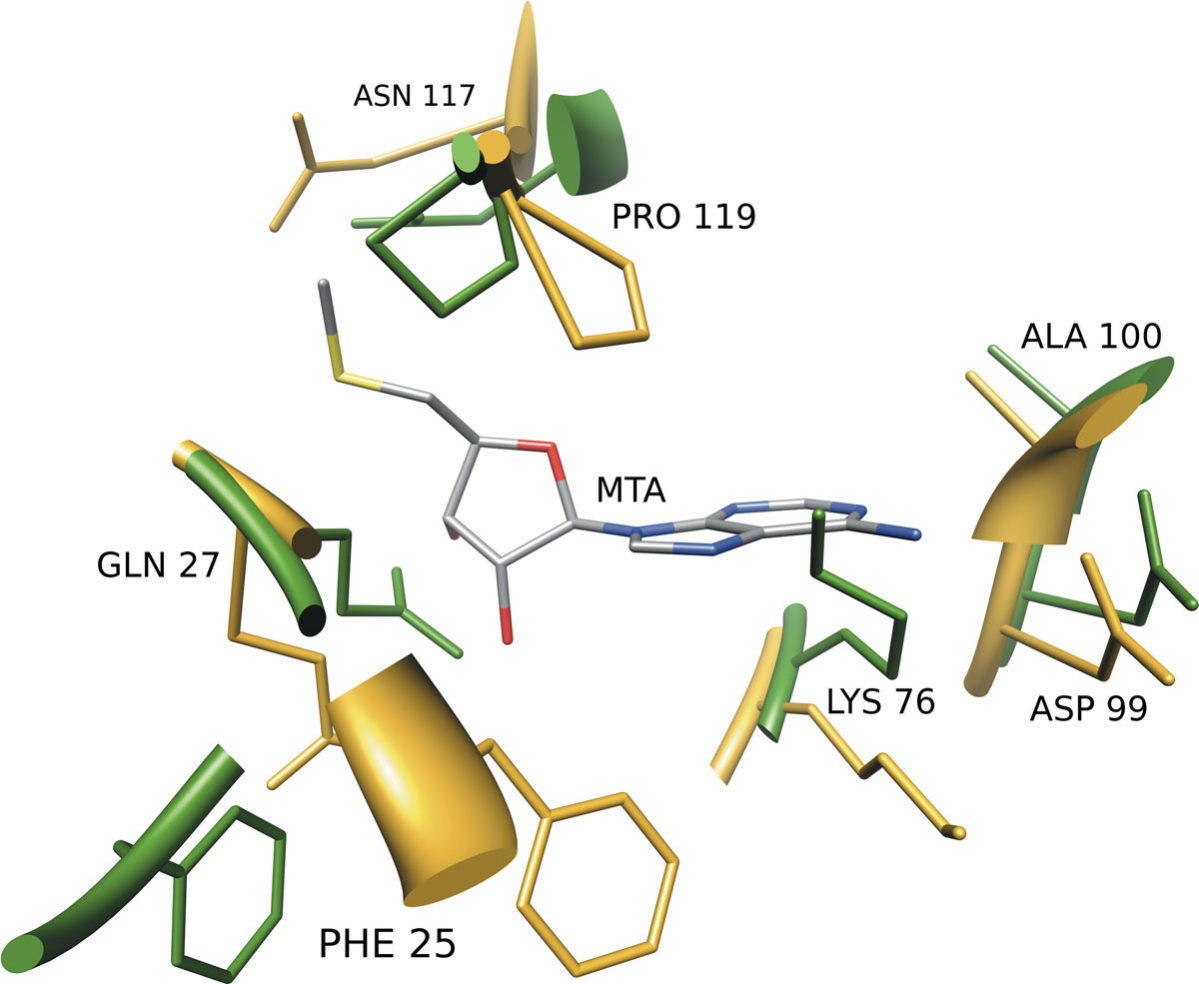
**PDBinder performance on the apo form of rRNA methyltransferase (KsgA)**. The binding site of the 16S rRNA methyltransferase (KsgA) from Thermus thermophilus in complex with 5'-methylthioadenosine (MTA). The holo form (PDB:3fut) is represented in yellow while the apo form (PDB:3fux) is in green. In order to evaluate the structural differences between the holo and apo forms, we superimposed the binding pocket residues (Residues 25, 27, 28, 54, 56, 75, 76, 99, 100, 117, 119) on their C-alpha atoms resulting in an RMSD of 2.05 Angstrom. Superimposed residues with an RMSD lower than 0.7 Angstrom are not represented in the picture (residues 54, 56, 75). PDBinder was able to identify all the binding residues of the holo form with the exception of Ala100 and Pro119. However, due to the high degree of conformational change, PDBinder did not identify residue Phe25 as binding in the apo form (RMSD 5.9). Even if the overall structure of the binding pocket is altered when the ligand is bound, the local conformation of small subsets of residues is mostly preserved and the method is able to identify seven of the eleven binding residues.

Using the Apo Test set of unbound structures the average MCC values calculated from the distribution was 0.271 with a sensitivity of 0.251, a specificity of 0.984 and a PPV of 0.372 (see Table 1). These results show that the performance of PDBinder suffers only a modest decrease when moving from holo to apo structures. This observation could be explained by the fact that our method considers the conformation of groups of three residues only. Therefore, even if the overall structure of the binding pocket is altered when the ligand is bound, the local conformation of small subsets of residues is mostly preserved (see Figure 4).

### Comparison of PDBinder with Q-SiteFinder

Q-SiteFinder is a method that scans the surface of a protein with a methyl probe and predicts clusters of favorable interaction spots as ligand binding sites. We developed an automated procedure to analyze the test set proteins using the Q-SiteFinder webserver [27]. In order to compare the results with those obtained with PDBinder, we had to map the cluster of probes predicted by Q-SiteFinder on specific residues of the structure. To this end we defined as binding residues those lying closer than 3.5 Angstrom to any cluster of probes identified by Q-SiteFinder.

We determined that Q-SiteFinder obtains the best performance in terms of MCC and PPV when the top two clusters are considered as positive predictions (data not shown) and applied this criterion in all the following analyses. The results obtained by both methods are displayed in Table 2. The performance of PDBinder on the holo set is better both in terms of PPV (0.413 vs 0.279) and MCC (0.313 vs 0.306). The sensitivity and specificity values show that PDBinder misses more true positives (sensitivity 0.295 vs 0.466) but makes more correct predictions on average (specificity 0.983 vs 0.934). Interestingly, while the performance of both methods as expected worsens when analysing the apo proteins, the relative decrease in performance of Q-SiteFinder is significantly greater. Indeed Q-SiteFinder suffers a 35% reduction in MCC vs 13% of PDBinder and a 28% reduction in PPV vs 10% of PDBinder.

**Table 2:** Results of integrating PDBinder with Q-SiteFinder

	SENS	SPEC	PPV	MCC	
**PDBinder (1)**	0.295	0.983	0.413	0.313	**HOLO**
**Q-siteFinder (2)**	0.466	0.934	0.279	0.306	
**(1) AND (2)**	0.154	0.997	0.483	0.245	
**(1) OR (2)**	0.622	0.913	0.286	0.365	

**PDBinder**	0.251	0.984	0.372	0.271	**APO**
**Q-siteFinder (2)**	0.324	0.931	0.200	0.199	
**(1) AND (2)**	0.098	0.997	0.375	0.168	
**(1) OR (2)**	0.497	0.909	0.231	0.280	

Integration of PDBinder with Q-SiteFinder. These results refer to the Apo/Holo Test Set. The first two rows detail the performance values achieved by PDBinder (1) and Q-SiteFinder (2) alone. The third row reports the results obtained if a residue is considered positive when it is predicted by both methods. The fourth row details the opposite situation in which a residue is considered positive when it is predicted by either method. The last row reports the results obtained with the two threshold systems. We performed a ten-fold cross validation to derive two different propensity thresholds, one for the residues predicted as positive by Q-SiteFinder and the other one for the negatives. The results are the average of the ten cross validation runs ± the standard error.

### Identification of the GDP binding pocket of the GDPRAN-NTF2 complex

**Figure 5:**
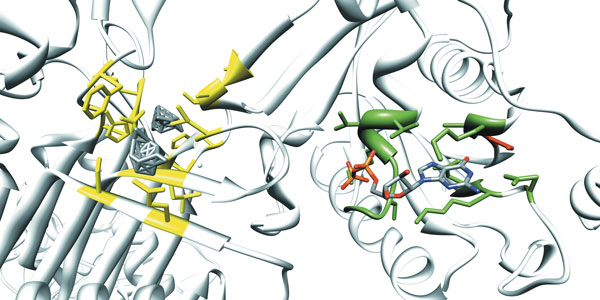
**Comparison of the results of PDBinder and Q-SiteFinder on the GDPRAN-NTF2 complex**. Side view of the GDPRAN-NTF2 complex (PDB: 1a2k) showing the A, B and D chains that surround the binding pocket. The residues correctly predicted as binding by PDBinder are in green while the false positives are in red. The grey shapes represent the top 2 ligand positions predicted by Q-SiteFinder. Residues that lie at less than 3.5 A from the Q-SiteFinder predictions are colored in yellow.

In order to illustrate the potential of PDBinder, we analyzed the GDPRAN-NTF2 complex (PDB: 1a2k) using both PDBinder and Q-SiteFinder. This complex has five chains, A and B forming the NTF2 protein and C D and E forming the GDPase Ran protein which binds a GDP molecule per chain. Figure 5 shows the predicted binding residues as sticks. The predictions of PDBinder are in green while those derived from the top 2 clusters of Q-SiteFinder are in yellow. PDBinder correctly identified all the binding site residues with only one false positive per chain, namely residue S150.

To understand why PDBinder achieved more accurate predictions on this structure with respect to an energy-based method, we analyzed in more detail the residues predicted as positives and negatives. All the residues identified by PDBinder as positives have a high number of matches with triplets found in binding pockets in the training set, and only one residue has a propensity value which is borderline but high enough to exceed the threshold of 0.143 (Table 3).

**Table 3:** Predicted binding residues for the GDPRAN-NTF2 complex

	CHAIN	RESIDUE	RESIDUE NUM	BIND	NON-BIND	PDBINDER PROPENSITY VALUE
**PDBinder predictions**	D	G	19	311	627	0.332
	D	G	20	409	487	0.456
	D	T	21	200	491	0.289
	D	G	22	868	754	0.535
	D	K	23	1112	267	0.806
	D	T	24	476	557	0.461
	D	T	25	418	535	0.439
	D	N	122	405	244	0.624
	D	K	123	381	156	0.709
	D	D	125	145	163	0.471
	D	S	150	227	433	0.344
	D	A	151	343	809	0.298
	D	K	152	31	175	0.150

**Q-SiteFinder predictions**	A	Q	65	1	109	0.009
	A	S	67	57	532	0.097
	A	Q	88	14	221	0.060
	A	L	89	24	1168	0.020
	A	I	96	9	313	0.028
	A	G	98	82	741	0.100
	A	A	122	16	538	0.029
	A	H	124	3	26	0.103
	B	M	97	8	207	0.037
	B	G	98	96	741	0.115
	B	A	122	19	568	0.032
	B	H	124	3	22	0.120
	B	F	126	0	10	0.000
	D	V	40	33	1820	0.018
	D	A	41	44	1033	0.041
	D	T	42	36	426	0.078

Binding site residues predicted by Q-SiteFinder and PDBinder on GDPRAN-NTF2 complex (PDB: 1a2k). The only false positive residue identified by PDBinder is S150.

The prediction of S150 as binding, the only false positive, may be explained by its structural neighbors raising the number of matches with the dataset of binding residues.

The analysis of the residues correctly predicted as negatives by PDBinder, and incorrectly marked as binding by Q-SiteFinder, shows the importance of considering geometry besides the simple relative abundance of a residue in binding pockets. Indeed for instance residue 124 of chain A is a histidine, which is 2.5-times more frequent in binding sites (Figure 3). However, in this case, this residue is in a different structural context and only has 3 matches against the entire set of binding residues. This histidine therefore has a low propensity value and is correctly predicted as negative.

### Integration with Q-SiteFinder

The approaches used by PDBinder and Q-SiteFinder are completely independent. The former is based on the analysis of the geometry of known binding residues, while the latter is based on predicting the energy of interaction with a chemical probe. We therefore decided to investigate whether a combination of the two approaches yielded improved results. To this end two different strategies were applied:

i) Predicting a residue as binding when at least one of the methods identifies it as such.

ii) Predicting a residue as binding only when both methods agree.

Table 2 displays the results of combining the two methods. When integrating the results using a simple boolean criterion (i.e. PDBinder AND/OR Q-siteFinder) the best performance is obtained by considering as positives the residues predicted by at least one method. With this approach there is a 0.052 (holo) and 0.009 (apo) increase in the MCC with respect to PDBinder alone but the PPV decreases by 0.127 (holo) and 0.141 (apo). Overall these changes are due to the fact there is a large increase in sensitivity accompanied by a smaller decrease in specificity, i.e. more of the true binding site residues are identified but the predictions are less correct on average.

### Comparison of PDBinder with other ligand binding site prediction methods

Large cavities in protein surfaces often harbor ligand binding sites or enzyme active sites, and several methods (e.g. SURFNET, LIGSITE) are available to identify such pockets. Mehio *et al*. recently developed a method called STP (Surface Triplets Propensities) that assigns to each residue of a structure a propensity value derived from counting the occurrences of triplets of atoms in known binding sites. The authors use these residue propensities to rank the surface cavities identified by SURFNET and then compare their results with those obtained with Q-SiteFinder and the method by Morita *et al*. The 35 apo proteins of the original Q-SiteFinder test set are used for the comparison.

**Figure 6:**
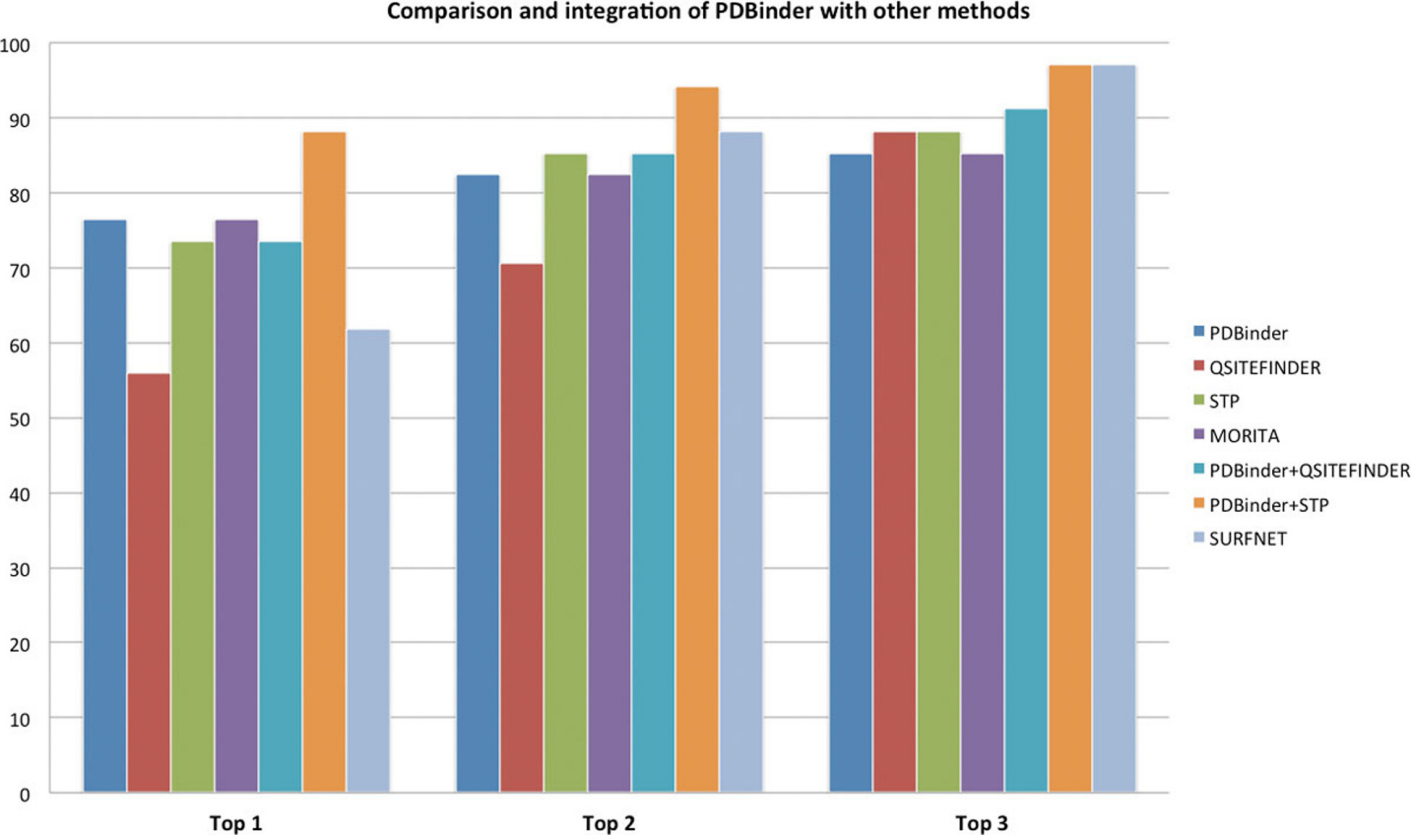
**Comparison with other binding site prediction tools**. Results obtained when applying different methods to the problem of ranking surface clefts identified by SURFNET to single out the correct ligand binding site. These data were obtained on a dataset of 35 apo proteins. The three different histograms report the number of correct identifications for each method when considering the top first, second and third predictions respectively.

We therefore decided to perform a similar experiment and used PDBinder to rank the cavities identified by SURFNET on the same dataset of 35 proteins. The ranking is based on the number of binding residues predicted by PDBinder in each cavity. Figure 6 displays the results of considering the top first, second or third-ranking cavities for the identification of the ligand binding site. The results show that PDBinder performs better than SURFNET, Q-SiteFinder, STP and the method by Morita *et al*. when only the first cavity is considered. Conversely when size alone is used for ranking SURFNET outperforms all the methods if one considers the top two or three cavities.

Similarly to what we did with Q-SiteFinder, we tried to integrate PDBinder and STP to investigate whether this led to a performance improvement. STP assigns a PatchScore to each atom and the authors consider 70 as the threshold for the identification of high scoring atoms which are then used to rank the cavities. For the purpose of integrating STP and PDBinder we consider any residue with at least one atom having a PatchScore greater than 70 as a binding residue. The number of residues predicted by either method is then used to rank the cavities.

As shown in Figure 6 the integration of PDBinder and STP clearly outperforms all the other methods, when only the top cavity is considered. When the top two cavities are considered PDBinder + STP outperforms SURFNET, while no single method does. Considering the top three cavities results in SURFNET and PDBinder + STP having identical performance and once again outperforming all the other methods.

## Conclusions

In this work we detailed the development of PDBinder, a novel method for predicting the location of ligand binding sites in protein structures. Our approach is based on the observation that unrelated binding sites contain similar structural motifs that are associated with specific chemical fragments in the ligands, irrespective of the identity of the ligand molecule as a whole. PDBinder therefore assumes that ligand binding sites will resemble each other more than other parts of the structure, because of the presence of these small motifs.

We built two libraries of binding and non-binding residues from a set of 1356 high-quality protein structures and used the Superpose3D local structural comparison algorithm to search for structural similarities between a query structure and the two libraries of residues. The number of matches with the sets of binding and non-binding residues is then used to assign a propensity value to each amino acid in the query structure. We optimized the score threshold on the training set of 1356 proteins using a leave-one-out procedure, and then tested PDBinder on the LigASite dataset, which comprises 239 apo/holo structure pairs.

We obtained an MCC of 0.313 on the holo set, with a PPV of 0.413. Interestingly the performance on apo structures is quite similar to the one obtained with the proteins in their ligand-bound conformation. This could be explained by the fact that, even though the ligand induces some rearrangements in the overall structure of the binding site, the local conformation of small sets of residues, which is the level of detail relevant for PDBinder, does not vary much. This result is extremely important for real-world applications where the location of the binding site is unknown because the protein has been crystallized without a ligand.

We then compared PDBinder with a number of existing methods. The comparison with Q-SiteFinder on the LigASite test set showed that PDBinder has a superior performance and this can be further improved by combining the two methods. We then used PDBinder to rank surface cavities identified by SURFNET and compared the performance with that of STP, Q-SiteFinder and the method by Morita *et al*. The results show that PDBinder is better than all other methods when considering only the first cavity as the correct one. Moreover combining PDBinder and STP outperforms all the other methods when the top ranking two or three cavities are also considered.

In conclusion this work shows that PDBinder is an efficient method for the prediction of ligand binding sites, that uses an approach which is very different from those found in the literature. The Propensity Value is a statistical potential unique among the other developed binding site prediction algorithms that not only has a good performance by itself, but also has an original approach which allows it to be integrated effectively with existing programs.

## Methods

PDBinder is a binding sites prediction method based on the assumption that the propensity of a residue to interact with a ligand is related to the number of times that the residue and its structural neighbours appear in the same geometric configuration in binding pockets of protein-ligand complexes of known structure. PDBinder uses the Superpose3D local structure comparison algorithm to identify local similarities between a query structure and two libraries of binding and non-binding residues respectively.

### The libraries of binding and non-binding residues

We derived two libraries of residues from all the proteins of known structure, one of residues in ligand binding pockets, and the other including all the remaining residues, according to the following procedure. We grouped together all the proteins in the Protein Data Bank having a sequence identity greater than 30% using the BLASTClust sequence clusters provided by PDB [28]. This initial set was composed of 144711 protein chains divided into 15376 homology groups. We decided to select only high-quality structures with a resolution lower than 2.0 Å inclusive and an R-factor lower than 0.20 Å inclusive. This reduced the set to 27399 chains divided into 4216 homology groups.

Binding pockets were defined by selecting all the residues having an atom closer than 3.5 Angstrom to any atom of a ligand. This distance threshold was determined during the training phase on our training set of proteins, after trying all the thresholds ranging from 3.0 to 10.0 Angstrom in increments of 0.5 (see Additional file 2: Table 2). We chose the value that resulted in the highest AUC.

Non-biological ligands present in crystallization buffers or solvents were excluded from the analysis [29]. We also removed metal ions, because they are often bound by few residues with strict geometric requirements [30], and ligands of extremely large size as shape complementarity is not effectively captured by our method.

Because of the above mentioned considerations only ligands having between 10 and 60 heavy atoms were included in the analysis. The lower bound of 10 excludes metal ions and very small molecules, while retaining ligands that take part in many enzymatic processes [8]. The upper bound of 60 heavy atoms led to the elimination of only 0.5% of the remaining ligands in the PDB. Protein chains that did not have any ligand fulfilling these criteria were removed from the analysis reducing the total to 14864 chains divided in 2062 homology groups. In order to make sure that the binding pockets in our dataset have comparable sizes we only kept the PDB chains that had at least 10 binding residues. This step reduced the number of chains in the dataset to 8444 divided into 1356 homology groups.

Only a single representative was retained for each homology group by selecting the chain with the highest number of residues located in a binding pocket. This was done to create a non-redundant dataset while at the same time preserving the greatest possible variability of binding pocket residues. The final dataset of 1356 protein chains was used to define the library of binding residues, i.e. the residues in a binding pocket, and non-binding residues, i.e. all the remaining amino acids. In total the binding dataset was composed of 1896 binding pockets comprising 25905 residues and the non-binding one of the remaining 423556 residues.

### Scanning a query structure with the libraries of residues

PDBinder uses Superpose3D, a fast local structure comparison method, to search for small (three residues) similarities between a query structure and the two libraries of binding and non-binding residues. Two sets of residues are considered similar if they can be superimposed with a Root Mean Square Deviation (RMSD) lower than a threshold. For this work we used an RMSD threshold lower than 0.7 Å as proposed in [31]. For the purpose of this analysis we only considered pairings between identical residues, i.e. no substitutions were allowed, to avoid having an excessively large number of matches. Additionally each residue in a match must be a neighbour (i.e. distance less than 7.5 Å) of at least another residue in the match, as defined in [25]. Given these constraints, the program uses an exhaustive depth-first search procedure to identify similarities between two sets of input residues.

Following the structural comparison we count the number of times each amino acid of the query protein is involved in a match with a group of residues from the binding (Tot_bind_) and non-binding (Tot_other_) datasets respectively.

The propensity value Pr is defined as:

$$\mathrm{Pr}=\text{To}{\text{t}}_{\text{bind}}/\text{To}{\text{t}}_{\text{bind}}+\text{To}{\text{t}}_{\text{other}}$$

**Figure 7:**
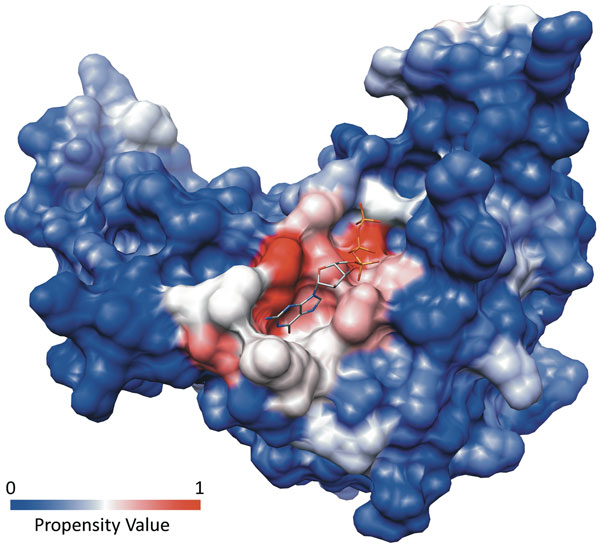
**Propensity values for the binding pocket of the GTPase from *Pyrococcus abissi *bound to GTP (PDB: **1yr8**) **. The surface of the protein has been colored according to the Propensity Value assigned to each residue by PDBinder. The colors range from blue, for low propensity values, to red for high values. The binding pocket clearly shows a strong positive signal.

This value ranges from 0, for residues that have never been involved in a match with a known binding pocket, to 1, for residues that are always found in matches with binding pockets. Figure 7 shows the surface of a structure colored according to the propensity values of the residues.

### Training set

We used the same non-redundant set of proteins from which we derived the binding and non-binding residue libraries to optimize the parameters of the method. Obviously, when deriving the propensity values for a protein chain, we exclude from the two libraries the residues coming from the chain itself (leave-one-out). Since the dataset is non-redundant (see above), when evaluating a structure from the training set, we compare it with binding pockets derived from proteins that have less than 30% sequence identity with the query structure.

### Test set

To test PDBinder we used LigASite, a golden standard dataset of biologically relevant ligand binding sites in protein structures. LigASite contains proteins with one unbound structure (apo) and at least one structure of the protein-ligand complex (holo). Correct quaternary structures suggested by PQS [32] were used to account for the fact that the quaternary arrangement of protein chains can influence the definition of the binding sites.

LigASite uses an automated approach to filter out non-relevant ligands based on their number of inter-atomic contacts with the protein. Redundancy is removed using the PISCES database [33] with a sequence identity cutoff value of 25%. PDB entries consisting of Cα traces were excluded from the list, as well as non X-ray entries, and X-ray entries with a resolution greater than 2.4 Å or an R-value greater than 0.25.

Starting from the non-redundant version of LigASite we created two datasets, one comprising apo-protein structures, and one comprising holo-protein structures. Since a single apo-protein may have more than one holo-protein associated, we created apo-holo pairs by retaining the holo protein which had the higher number of contacts with the ligand. Additionally, only pairs of proteins sharing 100% sequence identity were retained to allow an easy mapping of the binding residues from the holo to the apo structure.

Moreover we discarded 28 proteins in common with the Training set. The final test set consisted of 239 apo/holo pairs.

## List of abbreviations used

PDB: Protein Data Bank; STP: Surface Triplets Propensities; MCC: Matthew's Correlation Coefficient; PPV: Positive Predictive Value; AUC: Area Under the Curve; RMSD: Root Mean Square Deviation.

## Competing interests

The authors declare that they have no competing interests.

## Authors' contributions

VB developed the method, performed the statistical analysis, participated in the design of the study and drafted the manuscript. PFG participated in the design of the study and helped to draft the manuscript. MHC participated in the design of the study, in its coordination and helped to draft the manuscript. GA conceived the study, participated in its design and coordination and helped to draft the manuscript. All authors read and approved the final manuscript.

## Supplementary Material

Additional file 1 **Complete list of triplets along with their potentials**. For each triplet with the same amino acid composition we report the total number of matches with the library of binding (BIND) and non-binding (non-bind) residues. In the last column the average PDBinder Propensity Value is calculated for each triplet. For clarity, the row describing the behaviour of the VAL triplet reports the "bind" and "non-bind" data for all triplets containing a Val, an Ala and a Leu in any order and conformation. Click here for file

Additional file 2 **AUC by using different distance thresholds**. AUC achieved by PDBinder using different distance thresholds between the binding pockets residues and any atom of the bound ligand. Click here for file
