# Supplementary material for: Identification of binding pockets in protein structures using a knowledge-based potential derived from local structural similarities
Source: BMC Bioinformatics. 2012 Mar 28;13(Suppl 4):S17. doi: 10.1186/1471-2105-13-S4-S17 (PMC3434446; doi:10.1186/1471-2105-13-S4-S17)
Supplement: Additional file 2 — AUC by using different distance thresholds. AUC achieved by PDBinder using different distance thresholds between the binding pockets residues and any atom of the bound ligand. [file 1471-2105-13-S4-S17-S2.doc]

| **DISTANCE (Å)** | **AUC** |
| --- | --- |
| 3.0 | 0.737 |
| 3.5 | 0.765 |
| 4.0 | 0.726 |
| 4.5 | 0.719 |
| 5.0 | 0.717 |
| 5.5 | 0.713 |
| 6.0 | 0.708 |
| 6.5 | 0.702 |
| 7.0 | 0.694 |
| 7.5 | 0.689 |
| 8.0 | 0.685 |
| 8.5 | 0.683 |
| 9.0 | 0.681 |
| 9.5 | 0.678 |
| 10.0 | 0.677 |
